# Two-stage surgical reconstruction for cubitus valgus secondary to neglected lateral condylar fracture non-union in older children

**DOI:** 10.3389/fped.2026.1767262

**Published:** 2026-03-13

**Authors:** Yuan Xiao, Li-wei Xie, Chi Kang, Zhi-qiang Deng, Xin Liu, Yu-han Huang

**Affiliations:** Department of Pediatric Orthopedics, Sichuan Provincial Orthopedics Hospital, Chengdu, Sichuan, China

**Keywords:** children, cubitus valgus, lateral condylar fracture, non-union, two-stage reconstruction

## Abstract

**Background:**

This study aimed to evaluate the outcomes of a two-stage surgical protocol—comprising open reduction and internal fixation (ORIF) with bone grafting followed by corrective osteotomy—for managing cubitus valgus deformity secondary to neglected lateral condylar fracture non-union in older children.

**Methods:**

We retrospectively reviewed nine patients (5 males, 4 females; mean age, 11.1 years; range, 10–14 years) who underwent this two-stage procedure between January 2015 and January 2023. The mean interval from initial injury to first surgery was 7.7 years (range, 6–10 years). All patients presented with cubitus valgus deformity, ulnar neuropathy, and lateral elbow instability. The first stage involved ORIF via a lateral approach, thorough debridement of the non-union site, and filling of the bone defect with commercially available allogeneic or synthetic bone substitutes, stabilized with 4.0-mm cannulated screws and Kirschner wires. The limb was immobilized in a long-arm cast for three weeks, followed by active mobilization. The second stage—a medial closing-wedge supracondylar humeral osteotomy—was performed six months later and fixed with a reconstruction locking plate. Osteotomy hardware was removed six months postoperatively.

**Results:**

At a mean follow-up of 24.2 months (range, 16–35 months), all fractures achieved radiographic union. The mean preoperative carrying angle of 37.4° (range, 31°–45°) improved to a mean of 10.8° (range, 9°–14°), which is within the normal range and closely matched the contralateral side. Elbow range of motion was restored to near pre-injury levels, with no joint laxity or avascular necrosis of the lateral condyle. All symptoms of ulnar neuropathy resolved completely. Outcomes were rated as satisfactory in all patients according to the Flynn criteria, with no treatment failures.

**Conclusion:**

In this small retrospective series, the two-stage surgical strategy appears effective in addressing both mechanical instability from non-union and the functional/cosmetic deficits of cubitus valgus in older children with neglected lateral condylar fractures. However, given the limited sample size and relatively short follow-up duration, these preliminary findings warrant validation through larger prospective studies with longer-term observation.

## 1 Background

Lateral condylar fractures of the humerus in children are often subtle on initial radiographs, leading to potential neglect and misdiagnosis. Untreated or inadequately managed fractures frequently progress to non-union, which can result in progressive cubitus valgus deformity and chronic ulnar neuritis (1–5). Contributing factors include interposition of synovial fluid inhibiting fibrin clot formation, mechanical blockage by displaced fragments, persistent tensile forces from the forearm extensors, and the tenuous blood supply to the lateral condylar fragment (5–8).

The optimal management of established non-union with significant cubitus valgus in older children remains controversial (6–12). Some authors advocate against surgical intervention due to concerns regarding postoperative loss of elbow range of motion (ROM) and avascular necrosis (AVN) (6, 10). Conversely, others have reported favorable outcomes following surgical correction, typically using a single-stage procedure that combines non-union site debridement, internal fixation, corrective osteotomy, and occasionally anterior transposition of the ulnar nerve (11, 12).

However, we hypothesize that a one-stage approach carries a substantial risk of iatrogenic stiffness—particularly in elbows that retain functional mobility secondary to the pseudoarthrosis at the non-union site. To mitigate this risk, we adopted a two-stage strategy: the first stage focuses on achieving stable bony union and restoring elbow stability without aggressive soft-tissue dissection, while the second stage is dedicated to precise correction of the residual valgus deformity based on the functional outcome of the initial healing phase.

This study aimed to evaluate the clinical and radiographic outcomes of this staged surgical protocol for cubitus valgus secondary to neglected lateral condylar fracture non-union in older children. Our findings suggest that this approach may represent a viable alternative for complex, long-standing non-unions, effectively balancing the goals of biological healing, mechanical realignment, and preservation of elbow function—particularly in cases where single-stage reconstruction poses high risks of stiffness or AVN.

## 2 Methods

### 2.1 General data

This retrospective case series was conducted at Sichuan Provincial Orthopedic Hospital, a tertiary pediatric orthopedic referral center in Chengdu, China.

Inclusion criteria were: (1) age <14 years; (2) established lateral condylar fracture non-union (>12 weeks post-injury); and (3) cubitus valgus deformity ≥30°.

Exclusion criteria included: (1) prior surgical attempts at definitive fixation; (2) active infection at the time of presentation; and (3) incomplete medical records or loss to follow-up within 12 months after the second-stage surgery.

Follow-up duration was measured from the date of the second-stage reconstructive surgery to the patient's most recent outpatient visit (mean: 24.2 months; range: 16–35 months). All patients underwent standardized postoperative follow-up at 2 weeks (for wound assessment), 6 weeks, 3 months, 6 months, 12 months, and annually thereafter. At each visit, clinical evaluation included elbow range of motion, carrying angle measurement, ulnar nerve function, and pain assessment. Standard anteroposterior and lateral radiographs were obtained at every visit until radiographic union was confirmed.

Neurological assessment: Preoperative ulnar nerve dysfunction was retrospectively classified according to the McGowan grading system based on documented clinical findings. Grade I was defined as sensory symptoms (e.g., numbness or paresthesia in the ulnar nerve distribution) without motor deficit; Grade II included mild intrinsic muscle weakness (e.g., reduced grip strength, mild weakness of the fourth and fifth digits) without claw deformity or muscle atrophy; Grade III was characterized by overt claw hand, Froment's sign, and/or visible muscle wasting. No patients exhibited Grade III dysfunction (13).

This study was approved by the Ethics Committee of Sichuan Provincial Orthopedics Hospital. Informed consent was waived due to the retrospective nature of the study.

### 2.2 Surgical technique

#### 2.2.1 First stage: debridement, bone grafting, and ORIF

Under general anesthesia, the patient was placed supine with the affected arm abducted on an arm board. A tourniquet was applied after exsanguination. A standard lateral longitudinal incision was made, centered over the humeroradial joint. The non-union site was exposed through the interval between the triceps and brachioradialis, taking care to preserve the posterior soft-tissue hinge to maintain vascularity to the fragment. Fibrous tissue and sclerotic bone at the non-union interface were meticulously debrided until healthy, bleeding cancellous bone was encountered.

An attempt was made to achieve anatomic reduction under direct vision. In cases where anatomic reduction could not be achieved, the lateral condylar fragment was reduced with proximal translation to eliminate impingement on the radial head. Provisional fixation was achieved with Kirschner wires to ensure proper radiocapitellar alignment. Definitive fixation was accomplished using 4.0-mm cannulated lag screws, supplemented with smooth Kirschner wires if necessary. Residual osseous defects were filled with commercially available allogeneic, xenogeneic, or synthetic bone graft substitutes, selected based on institutional availability at the time of surgery.

Postoperatively, the limb was immobilized in a long-arm cast with the elbow at 90° of flexion and the forearm in neutral rotation for three weeks, after which active range-of-motion exercises were initiated.

#### 2.2.2 Second stage: corrective osteotomy

Six months after radiographic confirmation of union at the lateral condylar non-union site, a second-stage corrective osteotomy was performed. Preoperative planning was carried out on bilateral elbow extension anteroposterior (AP) radiographs. A medially based (closing-wedge) supracondylar osteotomy was templated parallel to the longitudinal axes of the humerus and forearm, with the target carrying angle individualized to match the contralateral side.

Through a proximal extension of the original lateral incision, the supracondylar osteotomy was performed. Intraoperative correction was verified using real-time fluoroscopy in combination with clinical assessment of elbow alignment and range of motion. The osteotomy site was stabilized with a lateral locking reconstruction plate, supplemented with smooth Kirschner wires when necessary. No postoperative external immobilization was applied, and active elbow mobilization was initiated on postoperative day two.

All procedures (both stages) were performed by a single senior pediatric orthopedic surgeon (Xin Liu, MD) with over 20 years of subspecialty experience in pediatric upper extremity reconstruction.

### 2.3 Outcome assessment

Outcomes were evaluated according to the Flynn criteria (14). Treatment was deemed a failure if any of the following occurred: (1) loss of elbow motion exceeding 15° compared to preoperative status; (2) residual carrying angle >15°; or (3) loss of forearm rotation exceeding 5°.

### 2.4 Statistical analysis

Given the small sample size and descriptive nature of this case series, no inferential statistical analyses were performed. Continuous variables are presented as mean ± standard deviation (SD) and range. Categorical variables are reported as frequencies and percentages. Data were managed and analyzed using Microsoft Excel.

## 3 Results

From January 2015 to January 2023, nine skeletally immature patients (5 males, 4 females; mean age, 11.1 years; range, 10–14 years) with established non-union of the lateral humeral condyle and concomitant cubitus valgus deformity were included in this study. Six patients had involvement of the left elbow and three of the right. The mean interval from initial injury to the first surgical intervention was 7.7 years (range, 6–10 years). This prolonged delay was primarily attributable to geographic and socioeconomic barriers: most patients originated from remote rural areas of Sichuan Province or neighboring regions (including parts of Tibet), where access to specialized pediatric orthopedic care is limited. Initial misdiagnosis, lack of caregiver awareness, and logistical challenges in reaching our tertiary referral center further contributed to late presentation.

The mean preoperative carrying angle was 37.4° (range, 31°–45°). When stratified by sex, male patients (n = 5) had a mean preoperative angle of 35.0° (range, 31°–39°), while female patients (*n* = 4) had a mean of 40.5° (range, 36°–45°). For reference, the mean carrying angle on the contralateral uninjured elbow was 10.2° (range, 6°–15°), serving as a patient-specific anatomical baseline. At final follow-up, the mean postoperative carrying angle improved to 10.8° (range, 9°–14°), with males averaging 9.6° (range, 9°–10°) and females 12.5° (range, 11°–14°). All postoperative angles fell within the respective normal ranges for sex (males: 5°–10°; females: 10°–15°) and were within ±4° of the contralateral side in every case.

All nine patients presented with preoperative ulnar neuropathy: five were classified as McGowan Grade I (sensory symptoms only), and four as McGowan Grade II (mild intrinsic muscle weakness without claw deformity or atrophy). At final follow-up, all patients demonstrated complete resolution of ulnar nerve symptoms, with no residual sensory or motor deficits. Lateral elbow instability was confirmed in all cases by a positive varus stress test (Table 1).

**Table 1:** Clinical and radiographic data of 9 patients.

Case	Gender	Age (y)	Side	Time since Injury (YS)	Contralateral Carrying Angle (°)	Preoperative Carrying Angle (°)	Postoperative Carrying Angle (°)	Preoperative Elbow ROM (°)	Postoperative Elbow ROM (°)	Preop. McGowan Grade	Follow-up (mo)
1	M	12	Left	7	10	35	9	123	112	I	30
2	F	14	Left	10	12	42	14	127	120	II	23
3	M	11	Left	6	8	38	10	124	131	I	16
4	F	13	Right	9	14	45	13	122	107	I	26
5	M	12	Left	8	7	31	9	126	120	I	16
6	F	14	Right	9	11	39	12	121	129	II	35
7	M	13	Left	7	9	32	10	128	116	II	24
8	F	10	Right	6	15	36	11	125	121	I	28
9	M	12	Left	9	6	39	10	124	117	II	20

All patients were followed for a mean of 24.2 months (range, 16–35 months) from the date of the second-stage osteotomy. Radiographic union of the lateral condylar non-union was achieved in all cases at a mean of 6.8 weeks (range, 4–10 weeks) after the first surgery. Following the second-stage osteotomy, all osteotomy sites healed radiographically at a mean of 7.2 weeks (range, 5–10 weeks). Elbow range of motion was restored to pre-injury levels in all patients.

**Figure 1:**
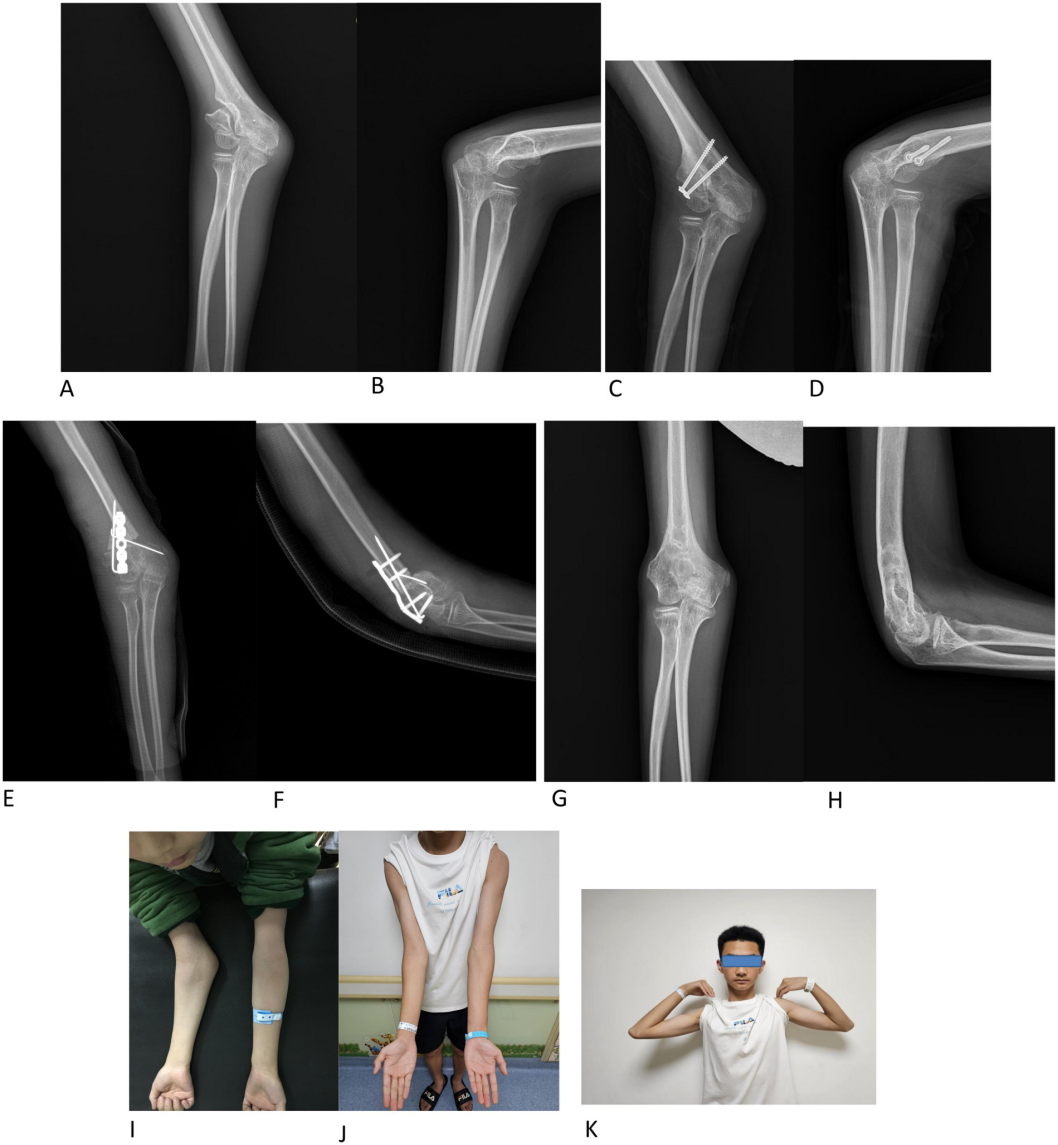
A 11-year-old with an old humeral condylar fracture of his right elbow. **A**–**B**: radiographs showing non-union of the old humeral condylar fracture and cubitus valgus. **C**–**D**: the first stage of surgery: internal reduction and use of 2 4.0 mm hollow compression screws to fix the block. **E**–**F:** the second stage of surgery: osteotomy of supracondylar humerus to correct cubitus valgus and internal fixation with a 5-hole reconstruction locking plate and 2 K-wires. **G**-**H**: radiographs one year after the second operation and after plate and K-wire removal. **I**: severe cubitus valgus before operation. **J**-**K**: normal appearance and function of the left elbow.

No complications were observed: there were no instances of delayed wound healing, surgical site infection, clinically significant heterotopic ossification (defined as ectopic bone formation resulting in functional impairment of the elbow), joint laxity, or radiographic signs of avascular necrosis of the lateral condyle. According to the Flynn criteria, clinical outcomes were rated as satisfactory in all patients, and there were no treatment failures. A representative case is illustrated in Figure 1.

## 4 Discussion

Chronic non-union of the lateral humeral condyle with established cubitus valgus deformity represents a formidable reconstructive challenge in pediatric orthopedics, often complicated by ulnar neuropathy and elbow instability (1–5, 7). This study demonstrates that a two-stage surgical strategy—comprising initial biological repair of the non-union followed by delayed corrective osteotomy—achieves 100% union, restores near-anatomic carrying angles, resolves all preoperative ulnar nerve symptoms, and yields uniformly satisfactory outcomes without treatment failure or major complications, even in patients with prolonged delays (mean 7.7 years) from injury to intervention.

Non-union of the lateral condylar fragment disrupts the integrity of the distal humeral articular surface, and subsequent malunion or asymmetric overgrowth can create a sloped trochlea that predisposes the elbow to lateral subluxation and instability (15–19). A critical determinant of success in such reconstructions is the preservation of the lateral condyle's precarious blood supply. Anatomical studies consistently show that epiphyseal vessels enter predominantly through the posterolateral non-articular surface; thus, maintaining the posterolateral soft-tissue hinge during exposure is essential to prevent iatrogenic avascular necrosis (AVN)—a complication notably absent in our series.

Our approach contrasts with recently reported single-stage techniques. He et al. (20) described a medial approach combined with trapezoidal osteotomy and bone grafting for similar deformities, achieving good angular correction and routinely performing ulnar nerve transposition. It should be emphasized that the medial approach itself does not directly compromise the vascular supply to the lateral condyle, as the dominant epiphyseal vessels arise posteriorly. However, because the non-union site resides on the lateral side, accessing and managing this lesion through a medial exposure requires indirect manipulation across the joint, which may inadvertently disturb the posterolateral soft-tissue attachments and introduce biological risk. In contrast, Tien et al. (16) advocated a posterior approach with dome osteotomy and *in situ* compression fixation without bone grafting, thereby preserving biology but necessitating triceps splitting—a maneuver known to impair the extensor mechanism and increase postoperative stiffness (19, 21). Both strategies address biological healing and mechanical realignment in a single stage, demanding high technical precision.

Our two-stage protocol uniquely decouples these objectives: Stage 1 utilizes a direct lateral approach to achieve debridement and stable fixation under clear visualization, fully preserving the posterolateral vascular envelope. Only after confirmed radiographic union (mean 6.8 weeks) is Stage 2 performed via proximal extension of the same lateral incision for patient-specific wedge osteotomy under fluoroscopic guidance. This staged philosophy not only avoids the indirect handling risks associated with medial exposure and the extensor disruption inherent to posterior approaches, but also accounts for our absence of AVN, heterotopic ossification, joint laxity, and treatment failures. Moreover, the complete resolution of ulnar neuropathy in all patients—without prophylactic nerve transposition—suggests that restoring native alignment alone may be sufficient to decompress the ulnar nerve in this specific pathoanatomy.

Although a posterior approach has been used successfully in some series (16), it typically requires splitting or detachment of the triceps tendon, which may compromise the extensor mechanism and increase the risk of postoperative stiffness (22). Our two-stage protocol, performed entirely through a lateral approach, avoids any disruption of the triceps and preserves elbow extensor function—particularly advantageous in adolescent patients.

### 4.1 Study limitations

This study has several limitations. First, the sample size was small, which limits statistical power and the generalizability of our findings. Second, the follow-up duration was relatively short (mean 24.2 months), precluding evaluation of potential long-term complications such as late-onset avascular necrosis, recurrence of deformity, or post-traumatic arthritis. Third, the absence of a control group—specifically, a cohort treated with a one-stage procedure—precludes a direct comparative analysis of the relative efficacy, functional outcomes, and complication profiles between these two surgical strategies.

In light of these limitations, we propose several directions for future research. Prospective, multicenter studies with larger cohorts are needed to validate the safety and reproducibility of this two-stage protocol across diverse patient populations. Additionally, randomized or matched-pair comparative trials against single-stage approaches would provide higher-level evidence regarding the optimal timing and staging of reconstruction in neglected lateral condyle non-unions. Finally, extended follow-up beyond five years is essential to assess the durability of correction and the incidence of degenerative joint changes into skeletal maturity.

## 5 Conclusions

Based on our experience in a small cohort of nine patients, a two-stage surgical strategy—comprising open reduction and internal fixation (ORIF) of the non-union followed by corrective supracondylar osteotomy—appears to be a promising approach for restoring elbow function and alignment in older children with established lateral humeral condyle non-union and secondary cubitus valgus deformity. However, given the limited sample size and relatively short follow-up duration (mean 24.2 months), these preliminary findings should be interpreted with caution. Larger prospective studies with extended follow-up are needed to confirm the long-term efficacy, safety, and comparative advantages of this two-stage protocol over alternative reconstructive strategies.

## Data Availability

The original contributions presented in the study are included in the article/Supplementary Material, further inquiries can be directed to the corresponding author.
